# Effects of exercise on mortality rates of individuals with severe mental illness

**DOI:** 10.3389/fpsyt.2022.907624

**Published:** 2022-10-04

**Authors:** David A. Baron, Asmita Mishrekar, Shan Kazmi

**Affiliations:** Western University of Health Sciences, Pomona, CA, United States

**Keywords:** exercise, morbidity and mortality, serious mental illness, treatment, health monitoring

## Abstract

Persons with severe mental illness have a 10-to-20-year shorter life span than the general public. Excess morbidity and mortality in this patient population has been described as a major public health challenge worldwide. Despite robust extant literature on the role of exercise in reducing morbidity and mortality, especially from cardiovascular disease and diabetes (highly prevalent in this patient population), Very few clinical programs or clinical research projects currently exist to implement and study the effects of exercise on decreasing morbidity and mortality in this highly vulnerable patient population. Given the global lack of trained mental health providers, the need to integrate healthcare providers from different disciplines, such as nurses, physical therapists, occupational therapists, physician assistants, cannot be overstated. This mini-review will provide an historic perspective and current data supporting the need to establish exercise, and other Lifestyle Psychiatry interventions, as a key component of treatment for all patients with serious mental illness.

## Introduction

The National Institute of Mental Health (NIMH) defines severe mental illness (SMI) as a “mental, behavioral, or emotional disorder resulting in serious functional impairment, which substantially interferes with or limits one or more major life activities” (1). Examples of SMI include schizophrenia, bipolar disorder, and schizoaffective disorder. These conditions can be debilitating for individuals and often create poor quality of life, ultimately resulting in shorter life spans, especially when accounting for disability-adjusted life years (2). Individuals with SMI die 10 to 15 years earlier than the general population, on average (3). Compared to healthy controls, SMI patients have a 1.5–2.6 times greater risk of death (4, 5). Mortality in patients with SMI can be attributed to suicide and accidents, but these are not the most common causes of death (6, 7). Cardiovascular, respiratory, infectious disease, diabetes mellitus, and cancers are major contributors to the excess mortality seen in patients with SMI (8). Cardiovascular disease is easily the highest among these and only 25% of those who died with SMI are given a diagnosis for this (9).

One approach to treating SMI would be to implement lifestyle interventions that would not only improve psychiatric symptoms, but also address underlying physical health conditions. These conditions, often brought on by morbid obesity secondary to many of the psychopharmacological treatments for SMI, later result in decreased life expectancy (3). One of the proposed mechanisms to increase life expectancy and reduce mortality, as well as improve quality of life, is physical exercise (10).

## Findings

As stated previously, the mortality rates are quite high in SMI patients and some studies even suggest that the mortality rate may be increasing (4). Exercise would be beneficial here as it improves mortality risk in most of the physical health conditions mentioned, particularly cardiovascular disease (11). There are additional reasons to consider exercise as a means of reducing mortality in SMI. For example, SMI patients tend to consume a poor diet compared to the general population that is high in saturated fat and low in fiber (12). SMI patients also live a more sedentary lifestyle compared to the general population and are more likely to be obese (13).

Antidepressant and antipsychotic medications are the mainstay of treatment options for SMI. However, obesity, hyperlipidemia, insulin resistance, and arrhythmias are well known side effects of these medications (14). Nonetheless, underdosing is not appropriate, as the psychiatric symptoms experienced in SMI are often debilitating. Exercise can be beneficial in this regard, as it can reduce the severity of psychiatric symptoms in a number of conditions. Most effects are seen in severe depression and anxiety (15). Improvement of SMI symptoms can be seen in insomnia, schizophrenia, dementia, delirium, and agitation (16–19). Medications are necessary as a baseline for treatment, but exercise can be a useful adjunct, not only for psychiatric disease but also for cardiovascular disease, as several studies have shown.

For example, a study was conducted on 51 overweight and obese individuals with SMI who underwent a 12-month multimodal weight control program. These individuals were all on a regimen of second-generation antipsychotic medications and divided into an intervention and control group. 31 out of the 51 individuals, called the intervention group, participated in a program that incorporated nutrition, exercise, and behavioral interventions. The remaining 20 individuals were in the control group and received antipsychotic treatment only. 20 out of the 31 subjects in the intervention group participated in the multimodal weight control program to its completion. Metrics used included body mass index, weight, hemoglobin A1c levels, blood pressure, and cholesterol levels. There were statistically significant improvements across all metrics in the intervention group, and those in the control group continued to gain weight. This research study suggests exercise can reduce disease burden and mortality (20).

## Discussion

The role of exercise in maintaining physical and mental health is not new. Its origins can be traced back to Hippocrates. Over the centuries, regular exercise has been demonstrated to not only help maintain health but be an effective component of a comprehensive biopsychosocial treatment strategy for virtually all chronic diseases. In fact, regular exercise has been shown to impact positive health outcomes from a biological, psychological, and social perspective. Despite the robust extant clinical literature on the role of exercise in promoting health and well-being, and decreasing morbidity and mortality, it has been largely neglected in patients suffering from serious mental illness, despite the shortened lifespan in this patient population noted above.

A reasonable question to consider is why is this effective treatment so infrequently included in the comprehensive treatment of patients suffering from serious mental illness? A number of factors may explain this ongoing issue. First, regular exercise has not been part of the “culture” of treating this patient population (21). Until the late 1970's, patients with SMI were treated in state hospitals for months to years. During these extended hospitalizations, patients would get daily activity and smoking breaks to get outside, but exercise was rarely included. As hospitalization stays continued to decrease to days to weeks over the past 40 years, structured exercise was even less, often restricted to smoke breaks in a confined locked area. Discharge planning was focused largely on psychotropic medication management, stable housing, and outpatient social support (22). The ongoing stigma of mental illness has likely played a role in this. Patients suffering from SMI have been portrayed as potentially dangerous and a burden to society on the big and little screen (movies and TV) over the years, and funding for research and treatment has lacked far behind other “physical” illnesses, like cardiac, endocrine and oncologic diseases.

From a purely public health perspective, including regular exercise in the treatment of patients suffering from SMI would likely result in significant cost savings, not to mention the positive impact on overall health, well-being and ultimately decreased morbidity and mortality. A key factor to consider in prescribing an exercise program should be consideration of what the patient finds enjoyable and is safe and feasible given their living situation (23). The increased socialization group exercise can provide, may also be an integral component of a comprehensive biopsychosocial treatment plan.

## Conclusion

The focus of this special issue of the journal was to examine strategies to decrease mortality in patients suffering from SMI. Although proper medication management and psychosocial intervention are important, the role of regular exercise cannot be overstated. The impact of Lifestyle Psychiatry interventions has been well documented to increase quality and quantity of life for all persons, especially those with chronic diseases (24). Exercise is a key component of Lifestyle interventions. We believe a thoughtful exercise plan should be included in every patient suffering from mental illness, especially those with SMI.

An exercise program has minimal side-effects, but should be monitored by a health professional, and is extremely cost-effective. Prospective outpatient, community-based clinical trials will help determine the most efficacious and effective interventions, from a personalized medical approach (25). Low tech, inexpensive treatments, like an exercise program, may have the greatest impact on increasing the lifespan in patients suffering with SMI. Let's have well controlled clinical data answer this question, never losing sight of the fact that it is one piece, albeit an important one of a larger treatment puzzle.

Although the role of regular exercise has been well documented to reduce mortality in persons with serious mental illness, the role of the psychiatrist has been largely neglected. As clinicians, psychiatrists providing care for those suffering from SMI must take an active role in “prescribing” and monitoring exercise as a critical component of comprehensive care of their patient. The extant literature does not emphasize the important role of engaging the patient to determine an exercise program that is logistically possible (access to equipment as needed), and most importantly something that is fun for the patient. Recent literature has reported the role wearable activity monitoring devices can play. Step tracking devices have been shown to increase walking distance daily through real time, non-judgmental feedback. Given the ever-growing clinical data on the value of exercise on physical and mental health for persons with SMI, we propose all psychiatry residency programs should include specific didactic and clinical instruction on how to incorporate personalized physical activity into the treatment plan for all patients with SMI, in the inpatient and outpatient setting.

## Author contributions

All authors listed have made a substantial, direct, and intellectual contribution to the work and approved it for publication.

## Conflict of interest

The authors declare that the research was conducted in the absence of any commercial or financial relationships that could be construed as a potential conflict of interest.

## Publisher's note

All claims expressed in this article are solely those of the authors and do not necessarily represent those of their affiliated organizations, or those of the publisher, the editors and the reviewers. Any product that may be evaluated in this article, or claim that may be made by its manufacturer, is not guaranteed or endorsed by the publisher.
